# Brainglance: Visualizing Group Level MRI Data at One Glance

**DOI:** 10.3389/fnins.2019.00972

**Published:** 2019-10-11

**Authors:** Johannes Stelzer, Eric Lacosse, Jonas Bause, Klaus Scheffler, Gabriele Lohmann

**Affiliations:** ^1^Tübingen University Hospital, Tübingen, Germany; ^2^Max Planck Institute for Biological Cybernetics, Tübingen, Germany; ^3^Max Planck Institute for Intelligent Systems, Tübingen, Germany

**Keywords:** fMRI, visualization, brain imaging, group analysis, single subject analysis

## Abstract

The vast majority of studies using functional magnetic resonance imaging (fMRI) are analyzed on the group level. Standard group-level analyses, however, come with severe drawbacks: First, they assume functional homogeneity within the group, building on the idea that we use our brains in similar ways. Second, group-level analyses require spatial warping and substantial smoothing to accommodate for anatomical variability across subjects. Such procedures massively distort the underlying fMRI data, which hampers the spatial specificity. Taken together, group statistics capture the *effective overlap*, rendering the modeling of individual deviations impossible – a major source of false positivity and negativity. The alternative analysis approach is to leave the data in the native subject space, but this makes comparison across individuals difficult. Here, we propose a new framework for visualizing group-level information, better preserving the information of individual subjects. Our proposal is to limit the use of invasive data procedures such as spatial smoothing and warping and rather extract regional information from the individuals. This information is then visualized for all subjects and brain areas at one glance – hence we term the method *brainglance*. Additionally, our method incorporates a means for clustering individuals to further identify common traits. We showcase our method on two publicly available data sets and discuss our findings.

## Introduction

With more than 25000 published studies, functional magnetic resonance imaging (fMRI) is a core method for studying the human brain. fMRI measures brain activity indirectly by detecting signal changes caused by the blood oxygen level dependent effect (BOLD) with a spatial resolution ranging from 1 to 4 mm and below. This allows to localize cognitive functions and to chart structure-function relationships. To map brain functions to brain areas, the *de facto* standard is to pool data across all participants and to compute statistics on the group level. However, there are several compelling reasons why such group-level inference is inherently problematic (Dubois and Adolphs, 2016). For instance, standard group-level inference relies on the similarity of spatial patterns of brain activation across subjects (Stelzer et al., 2014). In other words, they count upon a very similar functional topography within the population for a given task. Deviations from this fingerprint are considered to be noise. Therefore, group-level inference only assesses the effective overlap and is incapable to truthfully depict heterogeneity across subjects. This reflects negatively on the scientific inference process, exemplified in the following thought experiment: let us assume we scan 20 subjects with fMRI probing an experimental task. We further assume that five of the subjects rely on brain area X to perform the task, while this area is task-irrelevant for the rest of the group. We are now facing a dilemma when using conventional group-level inferences: either brain area X is labeled active or not active. In the first case, the activations of the small subset of subjects were sufficiently powerful to establish group-level involvement for area X. Given that 75% of the subjects did not require that brain area for the task we would make an incorrect scientific statement here. The second outcome fails to adequately describe the group, not deeming the brain area to be involved on the group level: a non-negligible portion of the subjects relied brain area X and we would draw an incomplete picture. In earlier work, we have quantified this responder vs. non-responder effect on group-level statistics using simulations (Stelzer et al., 2014). We found that scenarios like the one pictured above are realistic and likely wide-spread within the brain mapping literature. Unfortunately, such effects of heterogeneity are hidden and not accessible to researchers if standard group-level inference was applied.

From an empirical point of view, multiple studies found concerning levels of group-level heterogeneity in fMRI data: for instance, Miller et al. (Miller et al., 2009, 2012) found that individual memory-encoding activation patterns consistently reappear in a retest setting, however, are distributed heterogeneously within the group. Large individual variations have also been found for word encoding (Heun et al., 2000). Kherif et al. (2009) investigated the sources of inter-subject variability in a study on reading words aloud and found the main sources contributing to variability in subject age and cognitive strategy. Aquino et al. (2019) demonstrated that this issue is particularly severe when it comes to ultra-high-field fMRI, where individual response patterns are especially visible due to the increase in spatial resolution. The inter- and intra-subject variability in resting-state fMRI have been simultaneously charted by Chen et al. (2015) finding attention and somatomotor networks more stable and limbic, default, visual and control network to exhibit higher inter-subject variability. In summary, there is a growing body of literature connecting the occurrence and relevance of individual functional variations. Deviations from the effective group-overlap thus should not be dismissed as noise but rather should be incorporated for understanding of structure-function relationships.

In the field of psychology, recent trends embrace the importance of studying the individual thoroughly, confirming that group-level analyses may lead to inferential indeterminacy (Smith and Little, 2018). Functional relationships are located within the individual subject and not within a group-level abstraction. This statement is also true for brain functions; they are situated in the individual brain and its own dynamics, and not in an abstracted group level representation (Turner and De Haan, 2017).

Anatomical variability poses additional challenges for group-level inference, stemming from differences in morphology and folding patterns (Rademacher et al., 2001; Fischl et al., 2008; Stelzer et al., 2014). The differences become especially pronounced at smaller spatial scales, thus ultra-high resolution fMRI at 7T and above (Polimeni et al., 2018) are particularly affected. Voxel-wise correspondence of anatomical locations within a group thus appears unfeasible, which ultimately hampers the detection power. Let us assume that there is a large variation in terms of morphology and functional patterns within some brain area of interest and a voxel-wise matching is indeed not possible. Performing voxel-level group analysis may therefore fail to reveal the area as task-involved, simply because the active voxels do not overlap across subjects. To mitigate such variability, spatial smoothing is often employed. However, smoothing is no cure for this issue and rather creates further problems (Stelzer et al., 2014). Taken together, the combination of functional and anatomical variability impedes reproducibility. For instance, studies examining low number of participants suffer from poor reproducibility in task-based fMRI (Thirion et al., 2007; Griffanti et al., 2016; Poldrack et al., 2017), which may be partially explained by the above issues.

Currently, there exist only a few alternatives to conventional group-analysis in voxel space. The straightforward solution would be to analyze each subject separately in native space. However, not all subjects can be showcased in the reporting of findings as this would take too much space and be confusing. Apart from this, showing all subject’s brain maps at once makes it difficult to spot common patterns within the population. Rather, researchers handpick a set of representative subjects. Alternatively, the voxel-wise overlap of a group can be shown (Seghier and Price, 2016), visualizing the voxel-wise consistency across subjects. However, this is impractical for finding patterns of common activations within the group, unless the analysis is constrained to a region of interest.

Here, we propose an alternative method called *Brainglance* to visualize group-level MRI or fMRI data as a simple way to summarize inter-subject variability. Our method omits data deformations that come with spatial smoothing and warping to standard spaces, thus maximally preserves the original information. Instead, we warp an atlas to the individual subjects space and summarize the measure of interest in a region-by-region fashion. Our approach is agnostic to both the underlying atlas and data, allowing to display arbitrary types of data and their summary statistics on the group level. In the following, we will introduce our method and showcase two exemplary fMRI studies.

## Materials and Methods

We use two exemplary data sets for sake of illustration. We describe data postprocessing and atlas normalizations. Next, we will introduce our visualization scheme and algorithm and finish with a brief description of the clustering methods that can be optionally employed.

### Dataset 1: Midnight Scan Club

We used the Midnight scan club (MSC) data set (Gordon et al., 2017), as this study was comprised of ten subjects that were each scanned for ten times, yielding very stable brain patterns within individuals. We limited our analysis to the *resting state session*, which consisted of 818 volumes acquired with a volume TR of 2.2 s. The data was minimally preprocessed using fMRIprep (see above). Next, we computed Eigenvector centrality (Lohmann et al., 2010, 2018) maps (ECM) for each session. Eigenvector centrality maps summarize fMRI time series on basis of the first eigenvector, i.e., a 3D volume indicating the hierarchical connectivity within the network. Furthermore, we computed regional homogeneity (Zang et al., 2004) as complementary measure for local functional connectivity.

The results were then averaged subject-wise across all ten sessions.

### Dataset 2: Human Voice Areas

The second dataset we use is a large-scale study investigating individual differences in voice-sensitive brain areas (Pernet et al., 2015). Here, subjects were presented with vocal and non-vocal sounds. We chose this data set as it comprised a very large number of subjects, which were all scanned sufficiently long for stable single subject analysis, allowing to perform clustering. The data was minimally preprocessed using fMRIprep (see above). We then computed a General Linear Model, contrasting vocal minus non-vocal trials (lipsia *vwhiteglm*). The final output were subject-wise contrast maps indicating the *z*-scores of the GLM fit.

### Preprocessing

Results included in this manuscript come from preprocessing performed using *fMRIPrep* 1.3.1 (Esteban et al., 2019), which is based on *Nipype* 1.1.9 (Gorgolewski et al., 2011).

#### Anatomical Data Preprocessing

A total of 2 T1-weighted (T1w) images were found within the input BIDS dataset. All of them were corrected for intensity non-uniformity (INU) with N4BiasFieldCorrection (Tustison et al., 2010) distributed with ANTs 2.2.0 (Avants et al., 2008). A T1w-reference map was computed after registration of 2 T1w images (after INU-correction) using mri_robust_template (FreeSurfer 6.0.1, Reuter et al., 2010). The T1w-reference was then skull-stripped using antsBrainExtraction.sh (ANTs 2.2.0), using OASIS30ANTs as target template. Brain surfaces were reconstructed using recon-all (FreeSurfer 6.0.1, Dale et al., 1999), and the brain mask estimated previously was refined with a custom variation of the method to reconcile ANTs-derived and FreeSurfer-derived segmentations of the cortical gray-matter of Mindboggle (Klein et al., 2017). Spatial normalization to the *ICBM 152 Non-linear Asymmetrical template version 2009c* (Fonov et al., 2009) was performed through non-linear registration with antsRegistration (ANTs 2.2.0), using brain-extracted versions of both T1w volume and template. Brain tissue segmentation of cerebrospinal fluid (CSF), white-matter (WM) and gray-matter (GM) was performed on the brain-extracted T1w using fast (FSL 5.0.9, Zhang et al., 2001).

#### Functional Data Preprocessing

For each of the 1 BOLD runs found per subject (across all tasks and sessions), the following preprocessing was performed. First, a reference volume and its skull-stripped version were generated through co-registration with the OASIS template (Marcus et al., 2007) using fMRIprep. The BOLD reference was then co-registered to the T1w reference using bbregister (FreeSurfer) which implements boundary-based registration (Greve and Fischl, 2009). Co-registration was configured with nine degrees of freedom to account for distortions remaining in the BOLD reference. Head-motion parameters with respect to the BOLD reference (transformation matrices, and six corresponding rotation and translation parameters) are estimated before any spatiotemporal filtering using mcflirt (FSL 5.0.9, Jenkinson et al., 2002). BOLD runs were slice-time corrected using 3dTshift from AFNI 20160207 (Cox, 1996). The BOLD time-series, were resampled to surfaces on the following spaces: *fsaverage5*. The BOLD time-series (including slice-timing correction when applied) were resampled onto their original, native space by applying a single, composite transform to correct for head-motion and susceptibility distortions. These resampled BOLD time-series will be referred to as *preprocessed BOLD in original space*, or just *preprocessed BOLD*. The BOLD time-series were resampled to MNI152NLin2009cAsym standard space, generating a *preprocessed BOLD run in MNI152NLin2009cAsym space*. First, a reference volume and its skull-stripped version were generated using a custom methodology of *fMRIPrep*. Several confounding time-series were calculated based on the *preprocessed BOLD*: framewise displacement (FD), DVARS and three region-wise global signals. FD and DVARS are calculated for each functional run, both using their implementations in *Nipype* (following the definitions by Power et al. (2014). The three global signals are extracted within the CSF, the WM, and the whole-brain masks. Additionally, a set of physiological regressors were extracted to allow for component-based noise correction (*CompCor*, Behzadi et al., 2007). Principal components are estimated after high-pass filtering the *preprocessed BOLD* time-series (using a discrete cosine filter with 128 s cut-off) for the two *CompCor* variants: temporal (tCompCor) and anatomical (aCompCor). Six tCompCor components are then calculated from the top 5% variable voxels within a mask covering the subcortical regions. This subcortical mask is obtained by heavily eroding the brain mask, which ensures it does not include cortical GM regions. For aCompCor, six components are calculated within the intersection of the aforementioned mask and the union of CSF and WM masks calculated in T1w space, after their projection to the native space of each functional run (using the inverse BOLD-to-T1w transformation). The head-motion estimates calculated in the correction step were also placed within the corresponding confounds file. All resamplings can be performed with *a single interpolation step* by composing all the pertinent transformations (i.e., head-motion transform matrices, susceptibility distortion correction when available, and co-registrations to anatomical and template spaces). Gridded (volumetric) resamplings were performed using antsApplyTransforms (ANTs), configured with Lanczos interpolation to minimize the smoothing effects of other kernels. Non-gridded (surface) resamplings were performed using mri_vol2surf (FreeSurfer).

Many internal operations of *fMRIPrep* use *Nilearn* 0.5.0 (Abraham et al., 2014), mostly within the functional processing workflow.

Next, we temporally filtered the data using a high-pass with a cutoff frequency of 1/100 s (vpreprocess in lipsia 3.1.0). The MNI coregistered data was furthermore smoothed with Gaussian kernels with FWHMs of 3, 6 and 9 mm (vpreprocess in lipsia 3.1.0).

We used the resulting non-linear transformations and their inverse to coregister the atlas to the individual subjects. All analyses outside of fMRIprep were parallelized using *GNU parallel* (Tange, 2011).

### Atlas-Based Processing

Summarizing data across subjects requires a common reference system. Our method relies on the brain parcelation provided by an atlas and accommodates any atlas defined in volumetric space. Here, we chose the Brainnetome atlas (Fan et al., 2016), which consists of 210 cortical and 36 subcortical subregions, thus a total of *M* = 246 brain areas. For each of the *N* subjects, we warped the MNI-based atlas onto the individual anatomy by applying the inverse of the T1 to MNI warping as provided by fMRIprep. We used nearest-neighbor interpolation, ensuring integer values for the labels in native subject space. Next, we iterated through all *M* areas of the atlas and extracted the values (e.g., ECM, reho and GLM) of each individual subject for each of the *M* brain regions. We then averaged the voxel data for each brain area. Iterating over all subjects and brain areas yielded a matrix *S* of size *M × N*, where *S*_*ij*_ corresponds to the values within the *i*-th brain area of subject *j*.

### Brainglance Visualization

**FIGURE 1:**
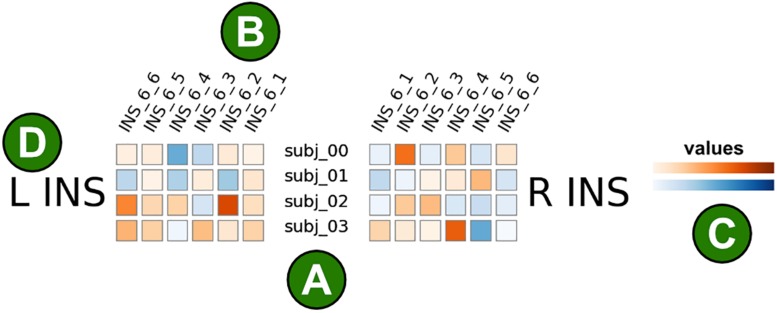
Illustration of the brainglance visualization. Each colored square corresponds to a single brain area from a single subject. **(A)** Subjects are shown in rows, here we show four subjects. **(B)** Brain areas are shown in columns. Here, six brain regions within the insular cortex are visualized. The brain areas are ordered in mirror-symmetric fashion such that the order of areas correspond to each other. The names of brain areas are shown in the abbreviated format as provided by the atlas description, here the Brainnetome atlas. For instance, INS_6_1 corresponds to the hypergranular insula, INS_6_2 the ventral agranular insula, INS_6_3 the dorsal agranular insula, INS_6_4 the ventral dysgranular and granular insula, INS_6_5 the dorsal granular insula and INS_6_6 the dorsal dysgranular insula. **(C)** The color corresponds to the measured value of interest, this could for instance be the strength of an activation. The color mapping is customizable, here we show positive values in orange and negative values in blue. **(D)** This figure only visualizes illustrative data from the insular cortex. The left insular cortex (L INS) is represented on the left half of the figure, the right insular cortex (R INS) on the right side.

The core idea of brainglance is to simultaneously depict all *N* subjects and all *M* brain areas at one glance. The underlying information is contained in the matrix *S*, as described above. Each brain area of each subject is then displayed in a square, the color indicating the given value found in the given brain area. This value can for instance be the mean activation level within the region, or any other summery statistics in scalar form. As the number of brain areas is large, the brain areas are shown grouped to their gross anatomical locations, e.g., grouping together frontal, temporal or parietal regions. We show the left hemisphere on the left side of the figure and the right hemisphere on the right. Within each gross region, we sorted the brain areas mirror in a symmetric fashion, so that the most inner brain areas for the left and right hemisphere correspond to each other. An example illustration is found in Figure 1.

### Clustering of Subjects

Our proposed method optionally includes clustering of the subjects. This can be useful if the subjects should be displayed in a grouped fashion, according to their similarity. Clustering may be employed if the number of subjects is very large and the data should be condensed for visualization. The starting point for the clustering procedure is a full single subject analysis for the entire group, represented as binary matrix *S* with size *M × N*, where *M* is the number of brain areas and *N* the number of subjects. Any clustering can be applied. Here, we chose the affinity propagation (AP) algorithm (Frey and Dueck, 2007), which uses the concept of message passing between the samples. AP has two main advantages: firstly, the identified clusters are a subset of the actual subjects thus the clustering approach attempts to find a representative subset and not an abstraction. Secondly, the number of does not have to be defined *a priori*. Both properties make affinity propagation clustering a viable approach and better suited as compared to other standard methods such as *k*-means clustering.

To get a qualitative impression of the differences in clustering, we averaged the 6 mm MNI data according to the clustering and show the results using the inflated MNI template for freesurfer.

### Statistical Evaluation Comparing Native Processing With MNI Space

We carried out further analyses to compare processing in native subject space (and coregistering the atlas to the individual anatomy as described above). For each subject and brain area, we subtracted the measured value (e.g., ECM, reho or GLM) in native space from the MNI warped results. This was done for all four smoothing levels for the MNI space data (0, 3, 6 and 9 mm). Next, we computed a one-sample *t*-test across all subjects for each individual brain area and thresholded the results at *p* = 0.05 (uncorrected). Furthermore we computed the mean difference across subjects for sake of visualization.

## Brainglance Software

This publication is based on the commit code.

e77c07fc7357cc60d1754481bb01b894a741620b.

### Installation

The software can be downloaded from a git repository found at:

https://github.com/lipsia-fmri/brainglance.

After cloning the repository (e.g., git clone^1^) the requirements need to be installed:

pip install -r requirements.txt

### Prerequisites

The following inputs are needed to run brainglance:

• A 3D brain map for each subject, containing results you want to display (preferentially in native subject space without smoothing). This could for instance be a GLM or ECM map.
• An atlas (e.g., Brainnetome, AAL,.). The atlas consists of a 3D nifty file and should be in the same space as the 3D brain map of each subject. Make sure to use nearest neighbor interpolation for any resampling steps involving the atlas, as the atlas 3D file should only include integer values.
• A description of the atlas. This description needs to be entered manually (see below). The description maps integer values found in the 3D atlas file to information that is necessary for display. Each occurring brain area in the 3D atlas file needs to be associated with the name of the region (“name_region”), the largescale name of the region (“name_largescale_region”) for sake of grouping (e.g., FRO, TEM,.) and lastly the hemisphere (can only be L and R). This type of information usually is supplied with every atlas.

### Running Brainglance

The first step is to instantiate the brainglance object:

bg = BrainGlance()

You will need to fill the description of the atlas. The “label” is corresponding to the integer value in the atlas. You can add all information manually, e.g., manually:

bg.add_atlas_definition_area(label = 1, name_area = "area1", name_largescale_region = "FRO", hemisphere = "L")

bg.add_atlas_definition_area(label = 2, name_area = "area2", name_largescale_region = "FRO", hemisphere = "L")

bg.add_atlas_definition_area(label = 3, name_area = "area1", name_largescale_region = "FRO", hemisphere = "R")

bg.add_atlas_definition_area(label = 4, name_area = "area2", name_largescale_region = "FRO", hemisphere = "R")

In case of the Brainnetome atlas, the following code achieves this:

M = 246

dp_atlas = ’/media/3tbd/studies/_data’

fn_atlas_descr = os.path.join(dp_atlas, ’BNA_brainatlas_areas.txt’)

atlas_descr = np.loadtxt(fn_atlas_descr, dtype = str,comments = ’#’,delimiter = ’\t’)

atlas_descr = atlas_descr[1:M + 1,:]

Next, we will read in this list into the brainglance object.

for i in range(M):

label = np.int(atlas_descr[i][0])

name_area = atlas_descr[i][1]

name_largescale_region = atlas_descr[i] [5].upper()

hemisphere = atlas_descr[i][2].upper()

bg.add_atlas_definition_area(label, name_area, name_largescale_region, hemisphere)

Now you need to add your subjects. For each subject, you need to provide the 3D brain map with values you want to show (fp_brainmap) and a coregistered atlas in native subject space (fp_atlas). Furthermore, you can supply the name of the subject as third argument, however, this is optional.

Add a single subject via

bg.add_subject(fp_brainmap, fp_atlas, "subject-01")

You can add more subjects with the same call:

bg.add_subject(fp_brainmap2, fp_atlas2, "subject-02")

bg.add_subject(fp_brainmap3, fp_atlas3, "subject-03")

Now you can generate a brainglance plot, supplying the figure output file *fp_figure*.

bg.draw_fingerprint(fp_figure)

## Results

In the following, we briefly describe visualization with our new method of the two example datasets: (1) the midnight scan dataset, where 10 individuals were scanned for 10 sessions each, and (2) the human voice area dataset, where 216 subjects had been scanned while listening to human voice versus control auditory stimuli.

### Midnight Scan Dataset

#### Eigenvector Centrality Mapping

**FIGURE 2:**
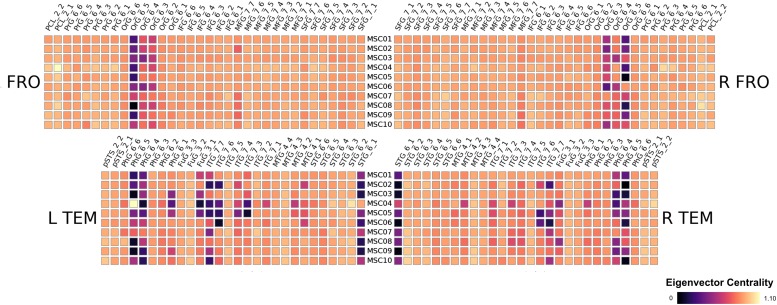
Results of the midnight-scan dataset, displaying the eigenvector centrality of the resting state scans (averaged over all sessions). We show only frontal and temporal regions here, the full results are shown in the supplements.

**FIGURE 3:**
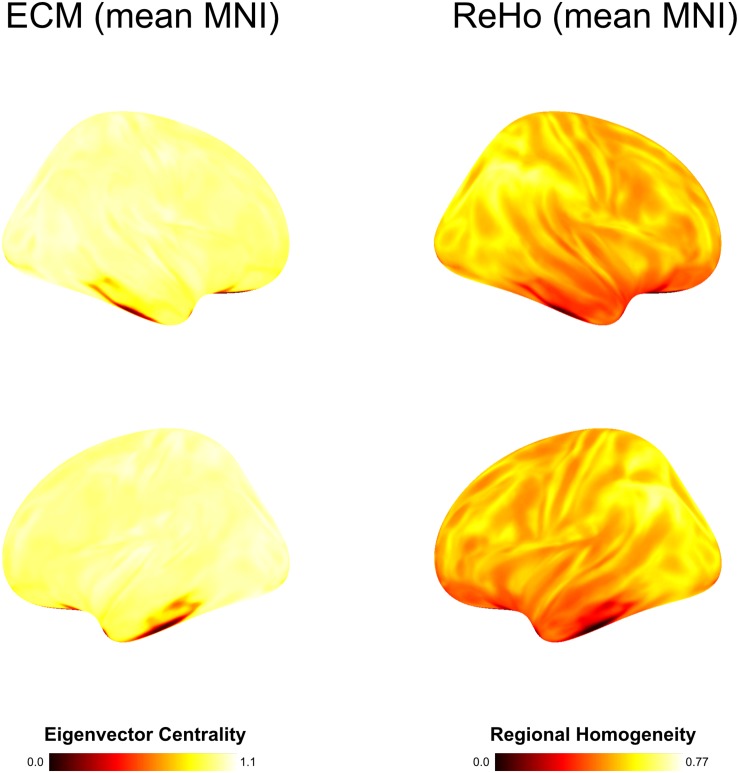
Group average computed on basis of MNI warped data with spatial smoothing of 6 mm. In contrast to individual subject-level analysis using brainglance, all inter-subject variability is lost.

We found striking similarities across subjects in their respective patterns of Eigenvector centralities. We show the results of the Frontal and Temporal Lobe in Figure 2 for the 10 subjects MSC01 to MSC10, note that the results are a subject-wise averages across 10 sessions. All subjects feature very low ECM values in the Orbital Gyrus (OrG 6_5, OrG 6_4 and OrG 6_3 bilaterally), the Parahippocampal Gyrus (PhG 6_5, PhG 6_4 bilaterally) and in the Superior Temporal Gyrus (STG 6_1 bilaterally). In contrast, subjects have larger variations in the Fusiform Gyrus (Fug 3_1) and the Inferior Temporal Gyrus (ITG 7_7, ITG 7_6, ITG 7_4 and ITG 7_3) and the in the Middle Temporal Gyrus (MTG 4_2). Our results largely follow conclusions from Gratton et al. (2018), highlighting large between-subject differences, which would be lost if subjects were averaged (see Figure 3).

Next, we compared the results from the single subject analysis in native space to analysis in MNI space after spatial smoothing. We used four different smoothing kernels (0, 3, 6 and 9 mm) for the MNI space data. We observed that multiple brain areas featured much larger ECM values in the smoothed MNI variants, most dominantly in bilateral PCL_2_1, FuG_3_2 and pSTS_6_2. On the other hand, several regions have much smaller ECM values when smoothing and MNI warping are used, e.g., in PhG_6_5, OrG 6_5, OrG 6_4 and Org 6_3. Thus, spatial smoothing and MNI warping modify the results in a spatially dependent matter. Furthermore, the differences are larger and more significant for 6 and 9 mm smoothing kernels.

#### Regional Homogeneity Analysis

Regional homogeneity analysis revealed striking differences across brain regions. Very high values were found bilaterally in the frontal area Org_6_5, and several temporal areas (PhG_6_5, PhG_6_4 and STG_6_1). While this overall pattern appears similar for all 10 subjects, there is also a large amount of variability across subjects for the given regions, rendering the practice of averaging across subjects questionable (see Figure 3).

Carrying out the regional homogeneity analysis in MNI space with spatial smoothing (with four smoothing kernels 0, 3, 6 and 9 mm) massively altered the results. Larger smoothing kernels resulted in higher values for reho (thus in a larger negative difference, as we computed native minus MNI processing). The result is not surprising, as spatial smoothing increases the local spatial correlation and thus homogeneity; therefore nearly all brain regions for all smoothing levels feature a significant change in reho. Interestingly, even without further spatial smoothing, the measured regional homogeneity in MNI space differed significantly from the native processed data.

### Human Voice Areas Dataset

**FIGURE 4:**
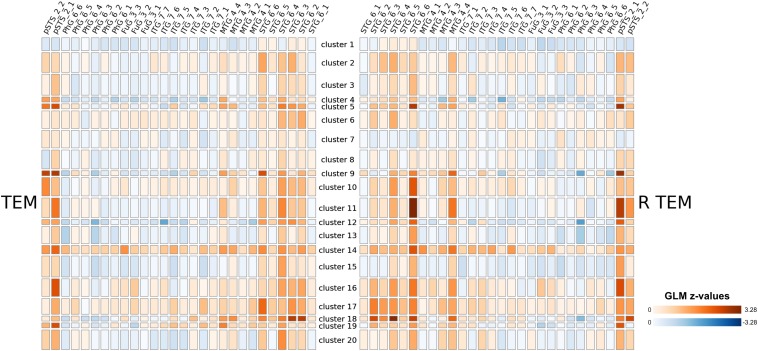
Results of a large-scale study involving 216 subjects that were presented auditory stimuli. Based on their GLM contrast subtracting non-vocal from vocal stimuli, we clustered the subjects into 20 clusters (as 216 subjects would be infeasible for visualization). The relative sizes of the resulting groups are indicated by the height of the group in the display, with logarithmic scaling. We show the full figure including all brain areas in the Supplementary Material. Note that the clustering procedure was based on whole-brain data.

**FIGURE 5:**
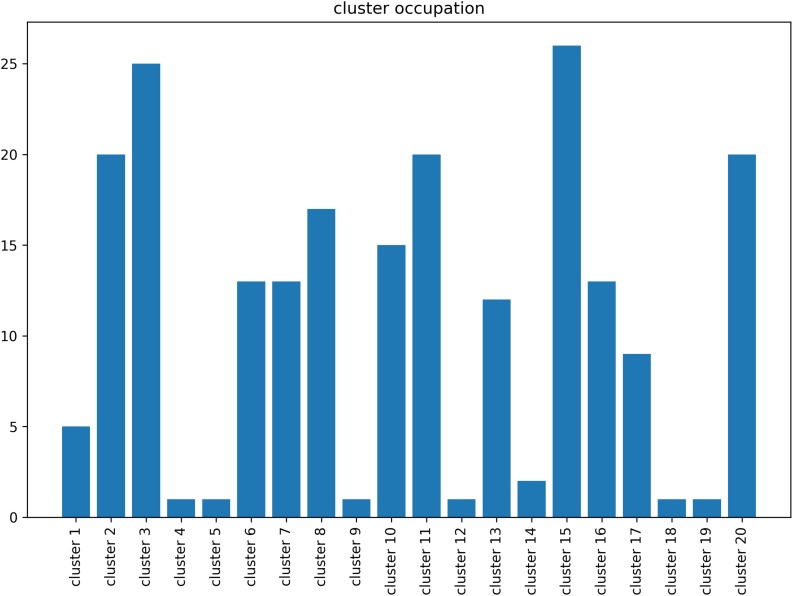
Size of the groups resulting from the clustering procedure of the human voice areas study. The largest group (cluster 15) contained 26 subjects, the smallest groups consisted of one individual subject (cluster 4, 5, 9, 12, 18 and 19).

In principal, brainglance would be capable to visualize the complete sample size of *N* = 216 subjects all at once. However, this amount of information might be overwhelming, therefore here we summarize the information by means of clustering, which also allows to detect sub groups in the cohort. The 216 subjects were separated into 20 subgroups, which we visualize in Figure 4. We show the distribution of group sizes in Figure 5. Note that the sizes of groups are also reflected in the brainglance plots in Figure 4, where the height corresponds to the logarithm of the group size.

From a coarse perspective, there are large overlaps and shared patterns across all groups, e.g., a strong bilateral activation in the Posterior Temporal Sulcus (pSTS 2_1 and pSTS 2_2). Furthermore, the Superior Temporal Gyrus shows strong activations across most groups, with exception of cluster 1, 7 and 8. More individual traits are visible in cluster 5, 9 and 11 in the right Posterior Temporal Sulcus (pSTS 2_1), which featured the strongest activation. Interestingly, cluster 14 showed large activations throughout the entire temporal cortex, while cluster 1 only featured very small activations here.

**FIGURE 6:**
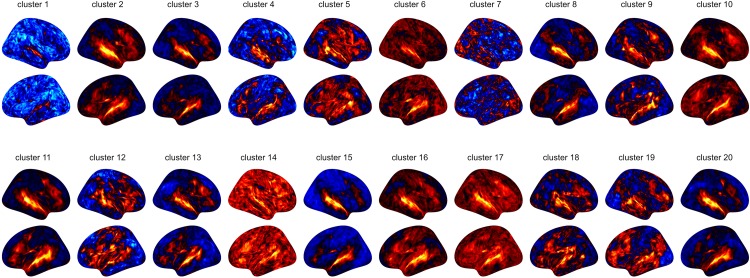
On basis of the clustering (taking place in native subject space), we averaged the GLM results of the voice areas study. Resulting mean images show substantial variation.

Given the clustering, we computed mean activation maps for each group on basis of the MNI warped 6 mm data, shown in Figure 6. While the temporal gyrus activation is shared across all subjects from a coarse perspective, there are substantial differences across the rest of the brain.

Furthermore, we assessed the impact of MNI warping and spatial smoothing for the voice areas study. Even without spatial smoothing, we found significant differences in activation strengths between native and MNI processing. For larger FWHMs, the difference was more pronounced; due to spatial smoothing *z*-values in the MNI maps were higher for regions particularly in the superior temporal gyrus (STG_6_2 to STG_6_6), but also in the middle temporal gyrus (MTG_4_4). The difference between 6 and 9 mm smoothing on the other hand is rather small.

## Discussion

**FIGURE 7:**
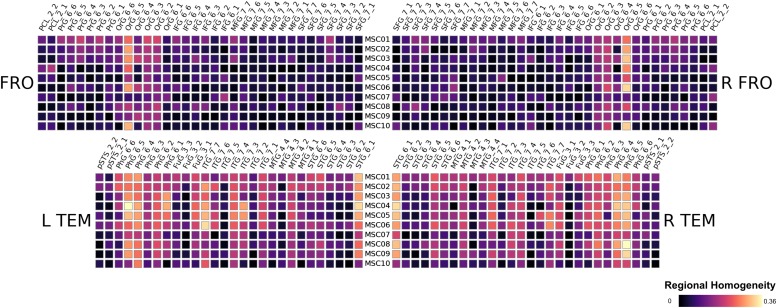
Results of the midnight-scan dataset, displaying the regional homogeneity of the resting state.

We present a new tool for visualizing group-level data for MRI and fMRI called *brainglance*. Our approach combines the strengths of both traditional group-level pooling methods and single-subject analysis: we preserve individual-level information without overwhelming researchers with a plethora of results from single subject brain map. Our approach targets to enable researchers to investigate individual patterns of brain activations and their relations within the group – all at one glance. Comparing the results of the commonly practiced group-level analysis to our *brainglance* method reveals that group-level procedures are only able to capture an effective overlap and ignore heterogeneity, as shown in Figures 2, 4, 7.

**FIGURE 8:**
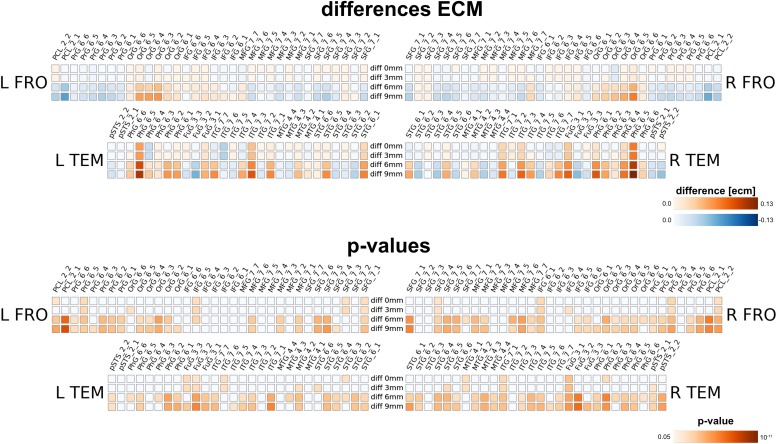
Comparison between analysis in native subject space versus MNI space with different smoothing FWHMs for eigenvector centrality mapping for four different smoothing levels (one per row), using 0, 3, 6, and 9 mm kernels. The upper panel shows absolute differences (native minus MNI), the lower panel shows a statistical evaluation of the differences.

**FIGURE 9:**
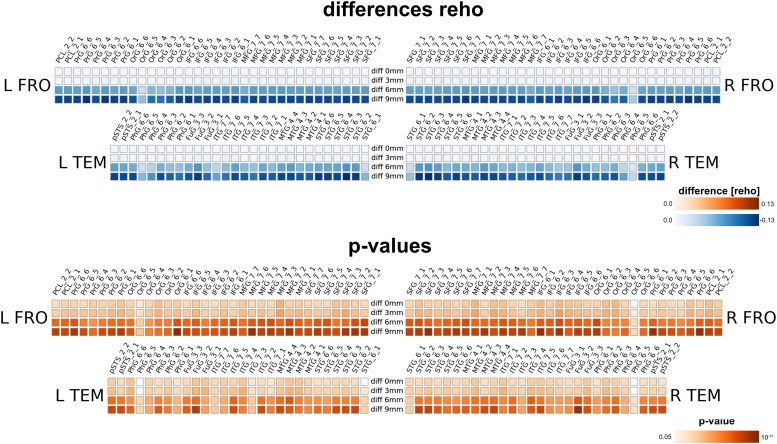
Comparison between analysis in native subject space versus MNI space with different smoothing FWHMs for regional homogeneity analysis. Larger smoothing kernels cause larger differences, however, there is already a substantial difference without further smoothing (0 mm) due to processing in MNI space.

**FIGURE 10:**
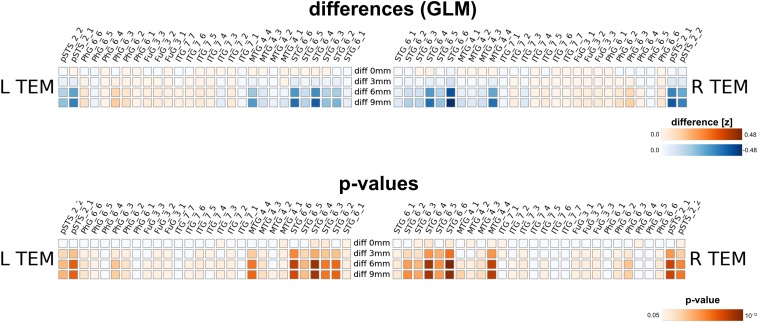
Comparison between analysis in native subject space versus MNI space with different smoothing FWHMs for the voice area study. While there are already changes in *z*-values without further spatial smoothing, the differences increased substantially for larger smoothing kernels.

Our method has several conceptual advantages over group-level averaging: no spatial smoothing and non-linear warping procedures distort the raw data, which remains in the native space of acquisition. Depending on the underlying measure, MNI warping and smoothing can massively alter the results, as shown in Figures 8, 9, 10. Interestingly, even without additional spatial smoothing, we found significant differences between native data processing (with the atlas warped to the individual) and warped data processing (taking the MNI atlas). As the difference itself depended on the underlying analysis method (i.e., ECM or reho, see Figures 8, 9) the difference is likely not due interpolation effects of warping the atlas to native space but rather stems from warping fMRI data to MNI space.

From a theoretical point of view, we want to point out that it is very desirable to avoid smoothing and thus mixing data across regions (e.g., consider signal washing from one hemisphere or gyri to the other). Furthermore, our method does not rely on a strict (and often unfeasible) voxel-level alignment of the activation peaks between subjects. Averaging over subjects on the voxel level may well cancel the activations if they are slightly offset. Thus, given anatomical variability and small-scale anatomical and organizational differences across subjects, voxel-wise averaging imposes biases, as more heterogeneous brain regions are subject to increased false negativity. The proposed *brainglance* method summarizes data on the level of a scalar summary statistic for each brain area. Therefore, heterogeneous spatial distributions of signal brain areas is accounted for and our approach avoids the above pitfalls.

Our method is flexible from a practical perspective: it can be employed for studies spanning only a handful of subjects but also for large-scale studies. In the latter case, we provide clustering methods to uncover common patterns of activation, stemming from different cognitive styles, genetic variability or environmental effects. Our approach is agnostic to the underlying data values: *brainglance* can display any type of data, ranging from task-based GLMs to network measures such as Eigenvector centrality, regional homogeneity and more. Furthermore, our method allows displaying any summary statistic representing brain areas, such as the mean, the variability, the maximum or minimum.

The Python-based software is distributed as an open- source repository on github: https://github.com/lipsia-fmri/brainglance. As open-source project, it enables further extensions, such as incorporating frameworks for statistical inference, multi-voxel pattern analyses or interactive browsing and display.

The main limitation of *Brainglance* is the dependency on accurate atlases. Warping standard atlases onto the individual brain does not warrant a correct anatomical delineation and may cause biases. It is worth stating our method is principally agnostic to the underlying atlas or segmentation. Here we chose the Brainnetome Atlas (Fan et al., 2016). The gold standard for deriving an anatomically realistic brain parcelation uses features from the individual micro- and macrostructure of the subject’s brain. Such *in vivo* Brodmann mapping approaches (Geyer et al., 2011) are the holy grail of quantitative neuroanatomy and show promising potential. They can be readily plugged into our method, boosting the spatial precision. Another alternative are individual segmentations that are derived manually from experts.

A further limitation is the dependency on correct coregistration to the individual subject. For instance, if there is a systematic shift in the registration, brainglance will reflect this error. Worst case, such shifts in registration will be falsely ascribed to functional variability, while they were indeed just registration errors. The smaller the brain area, the more problematic is this issue, as the relative count of misregistered voxels scales with the volume assuming the same shift. Furthermore, it also depends on the brainglance measure: the mean over each area might be considerably less sensitive to this issue than if only considering the minimum or maximum values. To mitigate this issue, we recommend the users to carefully check the coregistered atlases for accuracy and fix the registration, if problems are apparent.

Regarding the clustering procedure, we want to highlight a limitation with regards to the interpretation: on basis of our proposed method it is not possible to derive the underlying causal reasons for the subjects to fall into different subgroups. Reasons may range from large-scale differences in functional organization to effects that are due to differences in the individual neurovasculature and BOLD response strength. Thus, in the worst case, subjects have comparable neural responses but vastly different BOLD effects and thus would fall into different subgroups. The clustering provided by brainglance intends to provide a qualitative means for researchers to cluster their subjects and investigate whether this clustering is reflected in other, independent measures.

Abstracting data on the level of anatomical regions comprising hundreds of voxels may seem a suboptimal strategy due to the loss of spatial resolution. However, it should be common sense that group level analysis of fMRI *always* imply a loss in spatial specificity. While preprocessing steps such as non-linear registrations and spatial smoothing may preserve the nominal resolution, they drastically reduce the effective spatial resolution. After such operations, only a tiny fraction from the signal of the original spatial location is contained in a voxel (Stelzer et al., 2014), typically far below 10%. Overlaying such spatially imprecise results onto high resolution anatomical images falsely suggests an unrealistic spatial precision, and this practice should be considered misleading and scientifically unsound.

Future work will include various statistical tools for quantitative evaluation of individual differences to the group, which are outside the scope of the current visualization-based work. Such evaluations remain a great challenge, as individual-subject data often is lacks the statistical power for an appropriate statistical evaluation or test–retest reliability (Brandt et al., 2013).

## Data Availability

Publicly available datasets were analyzed in this study. This data can be found here: https://www.openfmri.org/dataset/ds000224/ and https://openneuro.org/datasets/ds000158.

## Ethics Statement

We are using publicly available data which we cite. All ethics protocols are in agreement with our local regulations.

## Author Contributions

JS designed and created the software. JS, EL, JB, KS, and GL wrote the manuscript.

## Conflict of Interest Statement

The authors declare that the research was conducted in the absence of any commercial or financial relationships that could be construed as a potential conflict of interest.

## Footnotes

1 https://github.com/lipsia-fmri/brainglance

## References

[B1] Abraham A. Pedregosa F. Eickenberg M. Gervais P. Mueller A. Kossaifi J. (2014). Machine learning for neuroimaging with scikit-learn. *Front. Neuroinform.* 8:14. 10.3389/fninf.2014.00014 24600388 PMC3930868

[B2] Aquino K. M. Sokoliuk R. Pakenham D. O. Sanchez-Panchuelo R. M. Hanslmayr S. Mayhew S. D. (2019). Addressing challenges of high spatial resolution UHF fMRI for group analysis of higher-order cognitive tasks: an inter-sensory task directing attention between visual and somatosensory domains. *Hum. Brain Mapp.* 40 1298–1316. 10.1002/hbm.24450 30430706 PMC6865556

[B3] Avants B. B. Epstein C. L. Grossman M. Gee J. C. (2008). Symmetric diffeomorphic image registration with cross-correlation: evaluating automated labeling of elderly and neurodegenerative brain. *Med. Image Anal.* 12 26–41. 10.1016/j.media.2007.06.004 17659998 PMC2276735

[B4] Behzadi Y. Restom K. Liau J. Liu T. T. (2007). A component based noise correction method (CompCor) for BOLD and perfusion based fMRI. *Neuroimage* 37 90–101. 10.1016/j.neuroimage.2007.04.042 17560126 PMC2214855

[B5] Brandt D. J. Sommer J. Krach S. Bedenbender J. Kircher T. Paulus F. M. (2013). Test-retest reliability of fMRI brain activity during memory encoding. *Front. Psychiatry* 4:163. 10.3389/fpsyt.2013.00163 24367338 PMC3856399

[B6] Chen B. Xu T. Zhou C. Wang L. Yang N. Wang Z. (2015). Individual variability and test-retest reliability revealed by ten repeated resting-state brain scans over one month. *PLoS One* 10:e144963-21. 10.1371/journal.pone.0144963 26714192 PMC4694646

[B7] Cox R. W. (1996). AFNI: software for analysis and visualization of functional magnetic resonance neuroimages. *Comput. Biomed. Res.* 29 162–173. 10.1006/cbmr.1996.0014 8812068

[B8] Dale A. M. Fischl B. Sereno M. I. (1999). Cortical surface-based analysis. I. Segmentation and surface reconstruction. *Neuroimage* 9 179–194. 10.1006/nimg.1998.0395 9931268

[B9] Dubois J. Adolphs R. (2016). Building a science of individual differences from fMRI. *Trends Cogn. Sci.* 20 425–443. 10.1016/j.tics.2016.03.014 27138646 PMC4886721

[B10] Esteban O. Markiewicz C. J. Blair R. W. Moodie C. A. Isik A. I. Erramuzpe A. (2019). fMRIPrep: a robust preprocessing pipeline for functional MRI. *Nat. Methods* 16 111–116. 10.1038/s41592-018-0235-234 30532080 PMC6319393

[B11] Fan L. Li H. Zhuo J. Zhang Y. Wang J. Chen L. (2016). The human brainnetome atlas: a new brain atlas based on connectional architecture. *Cereb. Cortex* 26 3508–3526. 10.1093/cercor/bhw157 27230218 PMC4961028

[B12] Fischl B. Rajendran N. Busa E. Augustinack J. Hinds O. Yeo B. T. T. (2008). Cortical folding patterns and predicting cytoarchitecture. *Cereb. Cortex* 18 1973–1980. 10.1093/cercor/bhm225 18079129 PMC2474454

[B13] Fonov V. S. Evans A. C. McKinstry R. C. Almli C. R. Collins D. L. (2009). Unbiased nonlinear average age-appropriate brain templates from birth to adulthood. *Neuroimage Suppl.* 1:S102 10.1016/S1053-8119(09)70884-70885

[B14] Frey B. J. Dueck D. (2007). Clustering by passing messages between data points. *Science* 315 972–976. 10.1126/science.1136800 17218491

[B15] Geyer S. Weiss M. Reimann K. Lohmann G. Turner R. (2011). Microstructural parcellation of the human cerebral cortex - from brodmann’s post-mortem map to in vivo mapping with high-field magnetic resonance imaging. *Front. Hum. Neurosci.* 5:19. 10.3389/fnhum.2011.00019 21373360 PMC3044325

[B16] Gordon E. M. Laumann T. O. Gilmore A. W. Newbold D. J. Greene D. J. Berg J. J. (2017). Precision functional mapping of individual human brains. *Neuron* 95 791–807.e7. 10.1016/j.neuron.2017.07.011 28757305 PMC5576360

[B17] Gorgolewski K. Burns C. D. Madison C. Clark D. Halchenko Y. O. Waskom M. L. (2011). Nipype: a flexible, lightweight and extensible neuroimaging data processing framework in python. *Front. Neuroinform.* 5:13. 10.3389/fninf.2011.00013 21897815 PMC3159964

[B18] Gratton C. Laumann T. O. Nielsen A. N. Greene D. J. Gordon E. M. Gilmore A. W. (2018). Functional brain networks are dominated by stable group and individual factors, not cognitive or daily variation. *Neuron* 98 1–20. 10.1016/j.neuron.2018.03.035 29673485 PMC5912345

[B19] Greve D. N. Fischl B. (2009). Accurate and robust brain image alignment using boundary-based registration. *Neuroimage* 48 63–72. 10.1016/j.neuroimage.2009.06.060 19573611 PMC2733527

[B20] Griffanti L. Rolinski M. Szewczyk-Krolikowski K. Menke R. A. Filippini N. Zamboni G. (2016). Challenges in the reproducibility of clinical studies with resting state fMRI: an example in early Parkinson’s disease. *Neuroimage* 124 704–713. 10.1016/j.neuroimage.2015.09.021 26386348 PMC4655939

[B21] Heun R. Jessen F. Klose U. Erb M. Granath D. O. Freymann N. (2000). Interindividual variation of cerebral activation during encoding and retrieval of words. *Eur. Psychiatry* 15 470–479. 10.1016/s0924-9338(00)00517-4 11175924

[B22] Jenkinson M. Bannister P. Brady M. Smith S. (2002). Improved optimization for the robust and accurate linear registration and motion correction of brain images. *Neuroimage* 17 825–841. 10.1006/nimg.2002.1132 12377157

[B23] Kherif F. Josse G. Seghier M. L. Price C. J. (2009). The main sources of intersubject variability in neuronal activation for reading aloud. *J. Cogn. Neurosci.* 21 654–668. 10.1162/jocn.2009.21084 18702580 PMC2766833

[B24] Klein A. Ghosh S. S. Bao F. S. Giard J. Häme Y. Stavsky E. (2017). Mindboggling morphometry of human brains. *PLoS Comput. Biol.* 13:e1005350. 10.1371/journal.pcbi.1005350 28231282 PMC5322885

[B25] Lohmann G. Loktyushin A. Stelzer J. Scheffler K. (2018). Eigenvector centrality mapping for ultrahigh resolution fMRI data of the human brain. *biorxiv* 1–8. 10.1101/494732

[B26] Lohmann G. Margulies D. S. Horstmann A. Pleger B. Lepsien J. Goldhahn D. (2010). Eigenvector centrality mapping for analyzing connectivity patterns in FMRI data of the human brain. *PLoS One* 5:e10232. 10.1371/journal.pone.0010232 20436911 PMC2860504

[B27] Marcus D. S. Wang T. H. Parker J. Csernansky J. G. Morris J. C. Buckner R. L. (2007). Open Access Series of Imaging Studies (OASIS): cross-sectional MRI data in young, middle aged, nondemented, and demented older adults. *J. Cogn. Neurosci.* 19 1498–1507. 10.1162/jocn.2007.19.9.1498 17714011

[B28] Miller M. B. Donovan C.-L. Bennett C. M. Aminoff E. M. Mayer R. E. (2012). Individual differences in cognitive style and strategy predict similarities in the patterns of brain activity between individuals. *Neuroimage* 59 83–93. 10.1016/j.neuroimage.2011.05.060 21651986

[B29] Miller M. B. Donovan C.-L. Van Horn J. D. German E. Sokol-Hessner P. Wolford G. L. (2009). Unique and persistent individual patterns of brain activity across different memory retrieval tasks. *Neuroimage* 48 625–635. 10.1016/j.neuroimage.2009.06.033 19540922 PMC2763594

[B30] Pernet C. R. McAleer P. Latinus M. Gorgolewski K. J. Charest I. Bestelmeyer P. E. G. (2015). The human voice areas: spatial organization and inter-individual variability in temporal and extra-temporal cortices. *Neuroimage* 119 164–174. 10.1016/j.neuroimage.2015.06.050 26116964 PMC4768083

[B31] Poldrack R. A. Baker C. I. Durnez J. Gorgolewski K. J. Matthews P. M. Munafò M. R. (2017). Scanning the horizon: towards transparent and reproducible neuroimaging research. *Nat. Rev. Neurosci.* 18 115–126. 10.1038/nrn.2016.167 28053326 PMC6910649

[B32] Polimeni J. R. Renvall V. Zaretskaya N. Fischl B. (2018). Analysis strategies for high-resolution UHF-fMRI data. *Neuroimage* 168 296–320. 10.1016/j.neuroimage.2017.04.053 28461062 PMC5664177

[B33] Power J. D. Mitra A. Laumann T. O. Snyder A. Z. Schlaggar B. L. Petersen S. E. (2014). Methods to detect, characterize, and remove motion artifact in resting state fMRI. *Neuroimage* 84 320–341. 10.1016/j.neuroimage.2013.08.048 23994314 PMC3849338

[B34] Rademacher J. Morosan P. Schormann T. Schleicher A. Werner C. Freund H. J. (2001). Probabilistic mapping and volume measurement of human primary auditory cortex. *Neuroimage* 13 669–683. 10.1006/nimg.2000.0714 11305896

[B35] Reuter M. Rosas H. D. Fischl B. (2010). Highly accurate inverse consistent registration: a robust approach. *Neuroimage* 53 1181–1196. 10.1016/j.neuroimage.2010.07.020 20637289 PMC2946852

[B36] Seghier M. L. Price C. J. (2016). Visualising inter-subject variability in fMRI using threshold-weighted overlap maps. *Sci. Rep.* 6:20170. 10.1038/srep20170 26846561 PMC4742862

[B37] Smith P. L. Little D. R. (2018). Small is beautiful: in defense of the small-N design. *Psychon. Bull. Rev.* 349 2083–2101. 10.3758/s13423-018-1451-8 29557067 PMC6267527

[B38] Stelzer J. Lohmann G. Mueller K. Buschmann T. Turner R. (2014). Deficient approaches to human neuroimaging. *Front. Hum. Neurosci.* 8:462. 10.3389/fnhum.2014.00462 25071503 PMC4076796

[B39] Tange O. (2011). *GNU Parallel - The Command-Line Power Tool. The USENIX Magazine.* Available at: usenix.org (accessed February 2011).

[B40] Thirion B. Pinel P. Mériaux S. Roche A. Dehaene S. Poline J.-B. (2007). Analysis of a large fMRI cohort: statistical and methodological issues for group analyses. *Neuroimage* 35 105–120. 10.1016/j.neuroimage.2006.11.054 17239619

[B41] Turner R. De Haan D. (2017). *Bridging the Gap Between System and Cell: The Role of Ultra-High Field MRI in Human Neuroscience*, 1st Edn. Amsterdam: Elsevier, 10.1016/bs.pbr.2017.05.005 28826512

[B42] Tustison N. J. Avants B. B. Cook P. A. Yuanjie Z. Egan A. Yushkevich P. A. (2010). N4ITK: improved N3 bias correction. *IEEE Trans. Med. Imaging* 29 1310–1320. 10.1109/TMI.2010.2046908 20378467 PMC3071855

[B43] Zang Y. Jiang T. Lu Y. He Y. Tian L. (2004). Regional homogeneity approach to fMRI data analysis. *Neuroimage* 22 394–400. 10.1016/j.neuroimage.2003.12.030 15110032

[B44] Zhang Y. Brady M. Smith S. (2001). Segmentation of brain MR images through a hidden Markov random field model and the expectation-maximization algorithm. *Med. Imaging IEEE Trans.* 20 45–57. 10.1109/42.906424 11293691

