# Supplementary material for: Brainglance: Visualizing Group Level MRI Data at One Glance
Source: Front Neurosci. 2019 Oct 11;13:972. doi: 10.3389/fnins.2019.00972 (PMC6797611; doi:10.3389/fnins.2019.00972)
Supplement: Supplementary file 1 [file Data_Sheet_1.PDF]

Supplementary Materials - Brainglance: visualizing group level MRI data at one glance

Supplementary Figure Midnight Scan Club

Results of the midnight-scan dataset, displaying the eigenvector centrality of the resting state scans (averaged over all sessions). Here, we show all brain regions simultaneously.

Supplementary Figure Voiceareas

Results of a large-scale study involving 216 subjects that were presented auditory stimuli. Based on their GLM contrast subtracting nonvoice from voice stimuli, we clustered the subjects into 19 groups. The relative sizes of the resulting groups are indicated by the height of the group in the display, with logarithmic scaling. Here, we show all brain regions simultaneously.

List of brain area abbreviations (Brainnetome Atlas)

**SFG_7_1**	Superior Frontal Gyrus	A8m, medial area 8
**SFG_7_2**	Superior Frontal Gyrus	A8dl, dorsolateral area 8
**SFG_7_3**	Superior Frontal Gyrus	A9l, lateral area 9
**SFG_7_4**	Superior Frontal Gyrus	A6dl, dorsolateral area 6
**SFG_7_5**	Superior Frontal Gyrus	A6m, medial area 6
**SFG_7_6**	Superior Frontal Gyrus	A9m,medial area 9
**SFG_7_7**	Superior Frontal Gyrus	A10m, medial area 10
**MFG_7_1**	Middle Frontal Gyrus	A9/46d, dorsal area 9/46
**MFG_7_2**	Middle Frontal Gyrus	IFJ, inferior frontal junction
**MFG_7_3**	Middle Frontal Gyrus	A46, area 46
**MFG_7_4**	Middle Frontal Gyrus	A9/46v, ventral area 9/46
**MFG_7_5**	Middle Frontal Gyrus	A8vl, ventrolateral area 8
**MFG_7_6**	Middle Frontal Gyrus	A6vl, ventrolateral area 6
**MFG_7_7**	Middle Frontal Gyrus	A10l, lateral area10
**IFG_6_1**	Inferior Frontal Gyrus	A44d,dorsal area 44
**IFG_6_2**	Inferior Frontal Gyrus	IFS, inferior frontal sulcus
**IFG_6_3**	Inferior Frontal Gyrus	A45c, caudal area 45
**IFG_6_4**	Inferior Frontal Gyrus	A45r, rostral area 45
**IFG_6_5**	Inferior Frontal Gyrus	A44op, opercular area 44
**IFG_6_6**	Inferior Frontal Gyrus	A44v, ventral area 44
**OrG_6_1**	Orbital Gyrus	A14m, medial area 14
**OrG_6_2**	Orbital Gyrus	A12/47o, orbital area 12/47
**OrG_6_3**	Orbital Gyrus	A11l, lateral area 11
**OrG_6_4**	Orbital Gyrus	A11m, medial area 11
**OrG_6_5**	Orbital Gyrus	A13, area 13
**OrG_6_6**	Orbital Gyrus	A12/47l, lateral area 12/47
**PrG_6_1**	Precentral Gyrus	A4hf, area 4(head and face region)
**PrG_6_2**	Precentral Gyrus	A6cdl, caudal dorsolateral area 6
**PrG_6_3**	Precentral Gyrus	A4ul, area 4(upper limb region)
**PrG_6_4**	Precentral Gyrus	A4t, area 4(trunk region)
**PrG_6_5**	Precentral Gyrus	A4tl, area 4(tongue and larynx region)
**PrG_6_6**	Precentral Gyrus	A6cvl, caudal ventrolateral area 6
**PCL_2_1**	Paracentral Lobule	A1/2/3ll, area1/2/3 (lower limb region)
**PCL_2_2**	Paracentral Lobule	A4ll, area 4, (lower limb region)
**STG_6_1**	Superior Temporal Gyrus	A38m, medial area 38
**STG_6_2**	Superior Temporal Gyrus	A41/42, area 41/42
**STG_6_3**	Superior Temporal Gyrus	TE1.0 and TE1.2

**STG_6_4**	Superior Temporal Gyrus	A22c, caudal area 22
**STG_6_5**	Superior Temporal Gyrus	A38l, lateral area 38
**STG_6_6**	Superior Temporal Gyrus	A22r, rostral area 22
**MTG_4_1**	Middle Temporal Gyrus	A21c, caudal area 21
**MTG_4_2**	Middle Temporal Gyrus	A21r, rostral area 21
**MTG_4_3**	Middle Temporal Gyrus	A37dl, dorsolateral area37
**MTG_4_4**	Middle Temporal Gyrus	aSTS, anterior superior temporal sulcus
**ITG_7_1**	Inferior Temporal Gyrus	A20iv, intermediate ventral area 20
**ITG_7_2**	Inferior Temporal Gyrus	A37elv, extreme lateroventral area37
**ITG_7_3**	Inferior Temporal Gyrus	A20r, rostral area 20
**ITG_7_4**	Inferior Temporal Gyrus	A20il, intermediate lateral area 20
**ITG_7_5**	Inferior Temporal Gyrus	A37vl, ventrolateral area 37
**ITG_7_6**	Inferior Temporal Gyrus	A20cl, caudolateral of area 20
**ITG_7_7**	Inferior Temporal Gyrus	A20cv, caudoventral of area 20
**FuG_3_1**	Fusiform Gyrus	A20rv, rostroventral area 20
**FuG_3_2**	Fusiform Gyrus	A37mv, medioventral area37
**FuG_3_3**	Fusiform Gyrus	A37lv, lateroventral area37
**PhG_6_1**	Parahippocampal Gyrus	A35/36r, rostral area 35/36
**PhG_6_2**	Parahippocampal Gyrus	A35/36c, caudal area 35/36
**PhG_6_3**	Parahippocampal Gyrus	TL, area TL (lateral PPHC, posterior parahippocampal gyrus)
**PhG_6_4**	Parahippocampal Gyrus	A28/34, area 28/34 (EC, entorhinal cortex)
**PhG_6_5**	Parahippocampal Gyrus	TI, area TI(temporal agranular insular cortex)
**PhG_6_6**	Parahippocampal Gyrus	TH, area TH (medial PPHC)
**pSTS_2_1**	posterior Superior Temporal Sulcus	rpSTS, rostroposterior superior temporal sulcus
**pSTS_2_2**	posterior Superior Temporal Sulcus	cpSTS, caudoposterior superior temporal sulcus
**SPL_5_1**	Superior Parietal Lobule	A7r, rostral area 7
**SPL_5_2**	Superior Parietal Lobule	A7c, caudal area 7
**SPL_5_3**	Superior Parietal Lobule	A5l, lateral area 5
**SPL_5_4**	Superior Parietal Lobule	A7pc, postcentral area 7
**SPL_5_5**	Superior Parietal Lobule	A7ip, intraparietal area 7(hIP3)
**IPL_6_1**	Inferior Parietal Lobule	A39c, caudal area 39(PGp)
**IPL_6_2**	Inferior Parietal Lobule	A39rd, rostrodorsal area 39(Hip3)
**IPL_6_3**	Inferior Parietal Lobule	A40rd, rostrodorsal area 40(PFt)
**IPL_6_4**	Inferior Parietal Lobule	A40c, caudal area 40(PFm)
**IPL_6_5**	Inferior Parietal Lobule	A39rv, rostroventral area 39(PGa)
**IPL_6_6**	Inferior Parietal Lobule	A40rv, rostroventral area 40(PFop)
**PCun_4_1**	Precuneus	A7m, medial area 7(PEp)
**PCun_4_2**	Precuneus	A5m, medial area 5(PEm)
**PCun_4_3**	Precuneus	dmPOS, dorsomedial parietooccipital sulcus(PEr)

**PCun_4_4**	Precuneus	A31, area 31 (Lc1)
**PoG_4_1**	Postcentral Gyrus	A1/2/3ulhf, area 1/2/3(upper limb, head and face region)
**PoG_4_2**	Postcentral Gyrus	A1/2/3tonIa, area 1/2/3(tongue and larynx region)
**PoG_4_3**	Postcentral Gyrus	A2, area 2
**PoG_4_4**	Postcentral Gyrus	A1/2/3tru, area1/2/3(trunk region)
**INS_6_1**	Insular Gyrus	G, hypergranular insula
**INS_6_2**	Insular Gyrus	vIa, ventral agranular insula
**INS_6_3**	Insular Gyrus	dIa, dorsal agranular insula
**INS_6_4**	Insular Gyrus	vId/vIg, ventral dysgranular and granular insula
**INS_6_5**	Insular Gyrus	dIg, dorsal granular insula
**INS_6_6**	Insular Gyrus	dId, dorsal dysgranular insula
**CG_7_1**	Cingulate Gyrus	A23d, dorsal area 23
**CG_7_2**	Cingulate Gyrus	A24rv, rostroventral area 24
**CG_7_3**	Cingulate Gyrus	A32p, pregenual area 32
**CG_7_4**	Cingulate Gyrus	A23v, ventral area 23
**CG_7_5**	Cingulate Gyrus	A24cd, caudodorsal area 24
**CG_7_6**	Cingulate Gyrus	A23c, caudal area 23
**CG_7_7**	Cingulate Gyrus	A32sg, subgenual area 32
**MVOcC _5_1**	MedioVentral Occipital Cortex	cLinG, caudal lingual gyrus
**MVOcC _5_2**	MedioVentral Occipital Cortex	rCunG, rostral cuneus gyrus
**MVOcC _5_3**	MedioVentral Occipital Cortex	cCunG, caudal cuneus gyrus
**MVOcC _5_4**	MedioVentral Occipital Cortex	rLinG, rostral lingual gyrus
**MVOcC _5_5**	MedioVentral Occipital Cortex	vmPOS,ventromedial parietooccipital sulcus
**LOcC_4_1**	lateral Occipital Cortex	mOccG, middle occipital gyrus
**LOcC _4_2**	lateral Occipital Cortex	V5/MT+, area V5/MT+
**LOcC _4_3**	lateral Occipital Cortex	OPC, occipital polar cortex
**LOcC_4_4**	lateral Occipital Cortex	iOccG, inferior occipital gyrus
**LOcC _2_1**	lateral Occipital Cortex	msOccG, medial superior occipital gyrus
**LOcC _2_2**	lateral Occipital Cortex	lsOccG, lateral superior occipital gyrus
**Amyg_2_1**	Amygdala	mAmyg, medial amygdala
**Amyg_2_2**	Amygdala	lAmyg, lateral amygdala
**Hipp_2_1**	Hippocampus	rHipp, rostral hippocampus
**Hipp_2_2**	Hippocampus	cHipp, caudal hippocampus
**BG_6_1**	Basal Ganglia	vCa, ventral caudate
**BG_6_2**	Basal Ganglia	GP, globus pallidus
**BG_6_3**	Basal Ganglia	NAC, nucleus accumbens
**BG_6_4**	Basal Ganglia	vmPu, ventromedial putamen
**BG_6_5**	Basal Ganglia	dCa, dorsal caudate
**BG_6_6**	Basal Ganglia	dlPu, dorsolateral putamen

**Tha_8_1**	Thalamus	mPFtha, medial pre-frontal thalamus
**Tha_8_2**	Thalamus	mPMtha, pre-motor thalamus
**Tha_8_3**	Thalamus	Stha, sensory thalamus
**Tha_8_4**	Thalamus	rTtha, rostral temporal thalamus
**Tha_8_5**	Thalamus	PPtha, posterior parietal thalamus
**Tha_8_6**	Thalamus	Otha, occipital thalamus
**Tha_8_7**	Thalamus	cTtha, caudal temporal thalamus
**Tha_8_8**	Thalamus	lPFtha, lateral pre-frontal thalamus

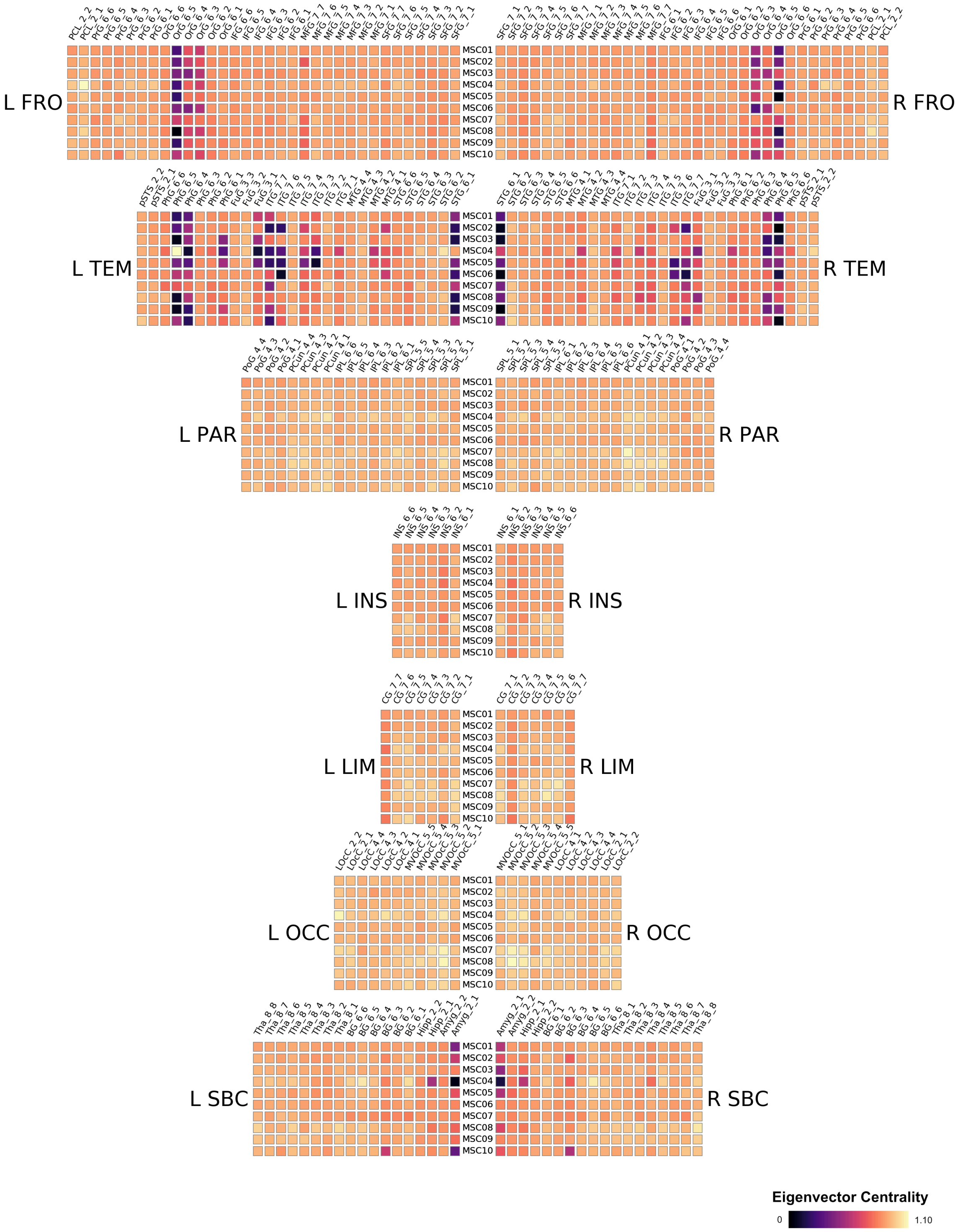

Supplementary Figure 1, MSC data, Eigenvector centrality.

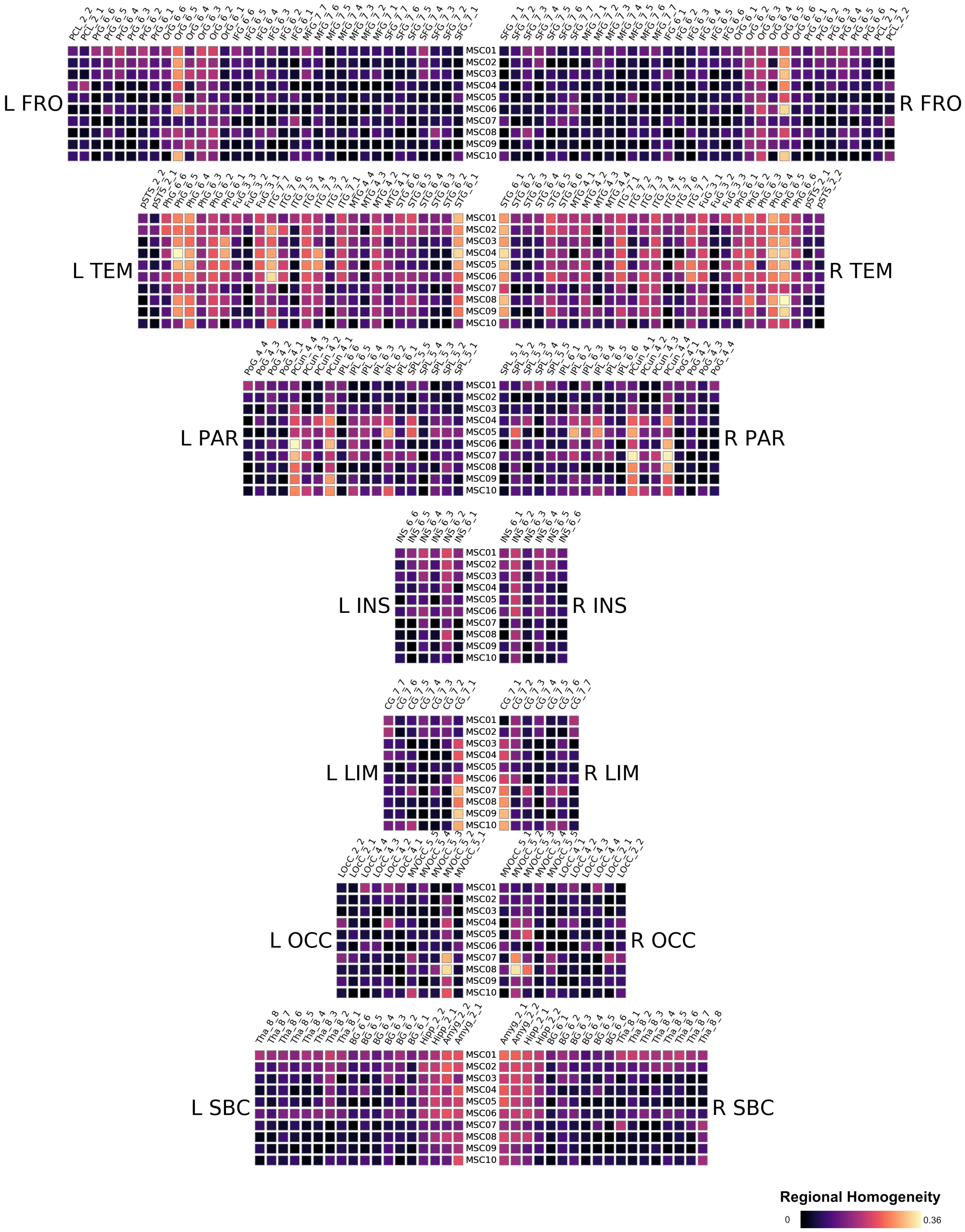

Supplementary Figure 2, MSC data, Regional Homogeneity

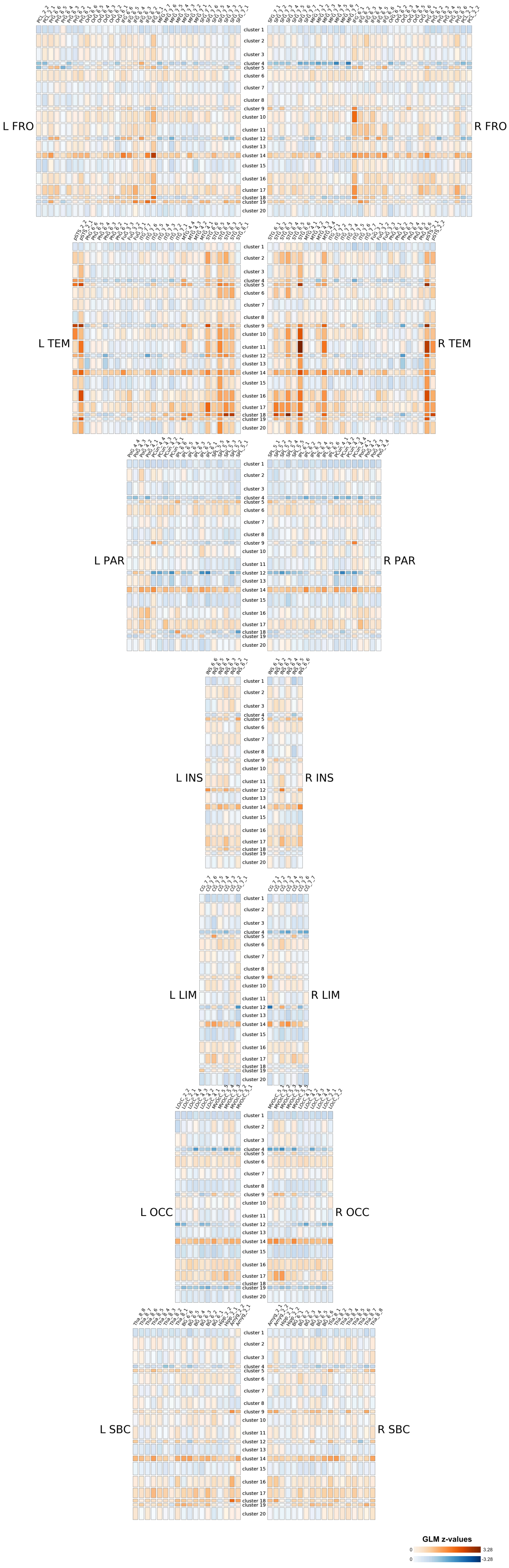

Supplementary Figure 3, Voice area dataset, GLM results

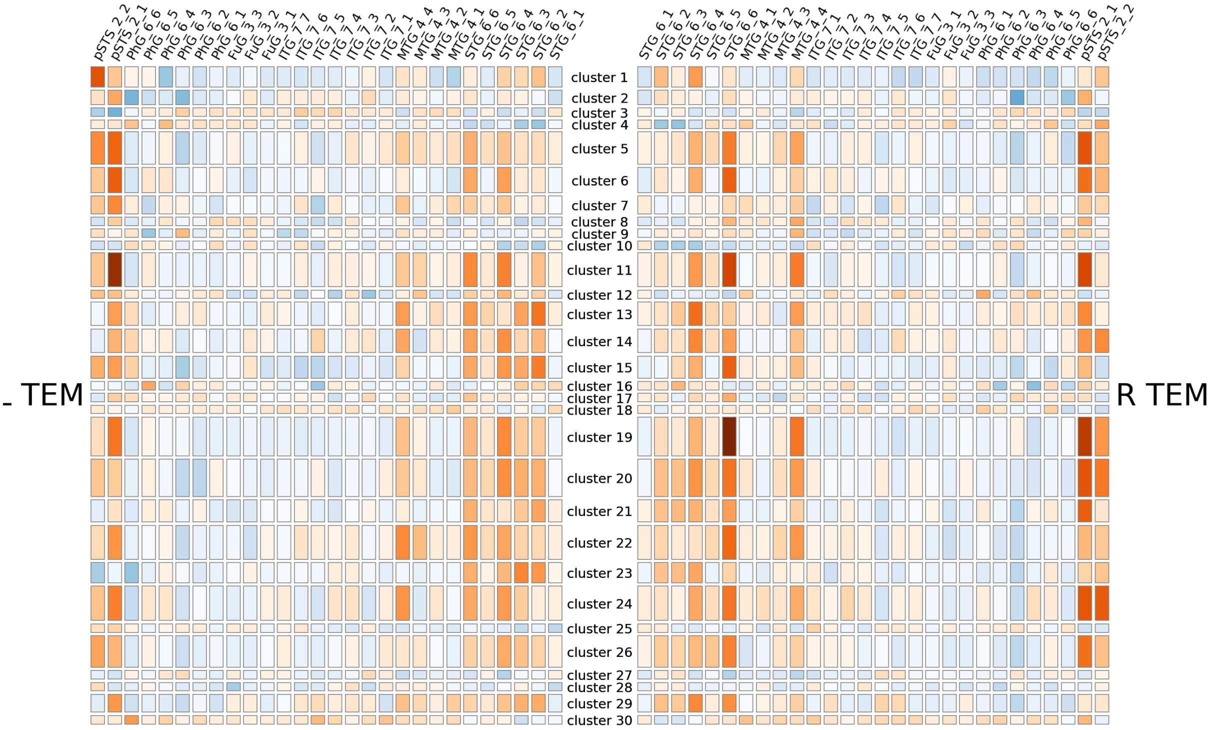

Supplementary Figure 4, Voice area study, where the intensities were normalized subject-wise *before* clustering was applied. The total number of clusters increased to 30 (from 20 without the normalization).
